# A Multidisciplinary Analytical Strategy for the Authentication and Quality Assessment of Bromelain in Dietary Supplements

**DOI:** 10.3390/ijms27093830

**Published:** 2026-04-25

**Authors:** Federico Benetti, Elena Petrini, Riccardo Sordi, Elisa Gaio, Marco Biagi

**Affiliations:** 1 ECSIN-ECAMRICERT SRL Laboratory (ECSIN), 35127 Padua, Italy; elena.petrini96@gmail.com (E.P.); r.sordi@ecamricert.com (R.S.); e.gaio@ecamricert.com (E.G.); 2 Department of Food and Drug, University of Parma, Parco Area delle Scienze 27/A, 43124 Parma, Italy; 3 SIFITLab, Italian Society of Phytotherapy, 53100 Siena, Italy; 4 Biopharmanet-TEC, Research Center for the Innovation of Health Products, University of Parma, 43124 Parma, Italy

**Keywords:** bromelain, adulteration, Western blot, enzymatic activity, food supplements, quality control

## Abstract

Bromelain is a complex of proteolytic enzymes obtained from *Ananas comosus*, widely used in dietary supplements for its anti-inflammatory, immunomodulatory, anti-edema, and wound-healing properties. Despite its broad commercial use, the quality control of bromelain-based products is often limited to enzymatic activity assays, which may not be sufficient for confirming authenticity. This study aimed to evaluate the quality of bromelain-containing products. Four commercially available bromelain samples were analyzed using a multidisciplinary analytical strategy. The study results demonstrated that only one of the assessed raw materials met the declared values in terms of bromelain activity and quality. Of the other three samples, two showed no enzymatic activity, while the other revealed activity complying with the declared specification that was due to papain, rather than bromelain. The present study demonstrates that enzymatic activity alone is insufficient to validate the quality of bromelain and related finished formulations. An integrated analytical approach is essential to detect adulteration, confirming authenticity and ensuring the safety and efficacy of bromelain supplements. Such methodologies should become a regulatory priority in the nutraceutical industry to protect consumer health and ensure product integrity.

## 1. Introduction

Bromelain is primarily extracted from the fruit and stem of pineapple (*Ananas comosus* (L.) Merr), a species belonging to the Bromeliaceae family. It constitutes an enzymatic complex including endopeptidases along with other enzymes such as acid phosphatase, glucosaminidase, peroxidase, and protease inhibitors [1]. Although bromelain is present in different parts of the plant, the highest concentration is found in the stem. Moreover, bromelain content can vary based on several factors, including growing conditions, country of origin, and the presence of different local cultivars [2]. Bromelain is widely recognized for its diverse biological properties, including anti-inflammatory, immunomodulatory, anti-edematous, and wound-healing effects, leading to its extensive use in dietary supplements [3]. In recent years, global health authorities, including the World Health Organization, have strongly emphasized the critical need for rigorous regulatory mechanisms and standardization to ensure the safety and quality of traditional and integrative medicines [4]. The standardization of botanical drugs requires comprehensive phytochemical, physico-chemical, and biological evaluations to guarantee a consistent chemical profile, batch-to-batch reproducibility, and global regulatory compliance [5,6,7]. Its biological effects and excellent safety profile are unequivocally linked to both the purity of bromelain and its source. Therefore, evaluating the quality of bromelain is particularly important; however, an in-depth approach is required due to its complex composition. While the quality of plant-derived products is typically assessed by characterizing their phytochemical profiles and secondary metabolites, this method is not applicable to bromelain. Instead, it is more appropriate to define its quality by evaluating its enzymatic activity—the functional core of the extract—alongside its specific protein composition. Notably, the bromelain used in the dietary supplement industry, accounting for over 90% of commercially available products, is typically qualified solely based on its enzymatic activity, and information regarding production quality and raw material purity is rarely provided [3,8]. Consequently, bromelain-based products are extremely heterogeneous and often exposed to the risk of adulteration. The efficacy and safety of bromelain-based products are closely related to the origin, purity, and enzymatic integrity of the raw material used. The hydrolytic activity against gelatin, expressed as Gelatin-Digesting Units (GDUs), is the most commonly used assay to qualify bromelain [9]. Despite the availability of various substrates ranging from gelatin, casein, and their nitro-derivatives to synthetic substrates such as Z-Arg-Arg-p-nitroanilide (pNA) and related compounds [10,11] to determine bromelain quality, the enzymatic activity test outcome can be affected by the presence of other proteases in the raw materials or bromelain-based product.

The adulteration of botanical ingredients represents a growing concern in the nutraceutical market. Multiple investigations and monitoring surveys, such as the Botanical Adulterants Prevention Program, have revealed several cases of adulteration of raw herbal materials [12]. These include both the intentional substitution of a declared ingredient with a cheaper or less effective one or the dilution of active constituents with inert or even harmful substances. Given its demand and the relatively high cost of its extraction under strict quality standards, bromelain is a product that is highly susceptible to adulteration. According to a recent survey conducted on samples from the US market, evidence of adulteration was detected in four batches of bromelain originating from India, with SDS-PAGE analysis revealing a complete absence of the characteristic bromelain bands. Furthermore, two out of three tested dietary supplements contained sulfur dioxide concentrations exceeding the specified limit of 10 ppm [13]. Moreover, the substitution of bromelain with other proteases such as papain or ficin, which have similar enzymatic activity but different biological specificity and origin, represents a subtle yet serious form of adulteration. Bromelain-containing dietary supplements must guarantee quantity, purity, and enzymatic activity corresponding to declared values, ensuring the absence of undeclared proteases such as papain or ficin. While enzymatic activity does not provide a complete picture of the overall quality of raw materials, quality controls should include the detection of biomarkers such as papain in order to adequately address the adulteration of bromelain-based products [8,12]. This emphasizes the urgent need for an integrated experimental approach to establish bromelain quality. For this purpose, more rigorous analytical methods that can distinguish authentic bromelain from adulterated or low-quality preparations need to be implemented by the manufacturers of effective and safe products. These methods should go beyond simple activity testing and adopt a broader and more comprehensive approach, including specific protein detection techniques capable of identifying unique molecular markers associated with bromelain from *A. comosus*.

The aim of this research is to develop a multidisciplinary analytical strategy for the authentication and quality assessment of bromelain used in dietary supplements, facilitating evaluation of the activity and purity of commercially available ingredients.

## 2. Results

### 2.1. Proteolytic Activity of Bromelain-Containing Ingredients

The enzymatic activity of four bromelain-based ingredients (raw materials) was measured spectrophotometrically using a specific chromogenic substrate and quantified by interpolating the absorbance with a calibration curve prepared with an FIP (International Pharmaceutical Federation) standard. Table 1 reports the enzymatic activity results in GDU/g, showing that while BROMELAIN 1 and 2 matched their expected nominal activity (with recoveries of 110.8% and 123.1%, respectively), BROMELAIN 3 and 4 exhibited no significant activity.

Bromelain contains a complex mixture of proteolytic enzymes, primarily cysteine proteases (also known as sulfhydryl proteases) of the C1A family such as papain-family proteases. As the chromogenic substrate has a similar affinity for both bromelain and papain (Km ≈ 0.34 mM for papain versus ~0.30 mM for bromelain) [14], the possible presence of other proteases could impact the determination of bromelain activity. To characterize the bromelain-based ingredients in terms of purity, bromelain activity was measured in the presence of increasing concentrations of antipain dihydrochloride, a specific papain inhibitor. Although this inhibitor reversibly inhibits serine/cysteine proteases and some trypsin-like serine proteases, its IC_50_ values depend on the protease being tested, with the highest affinity for papain. To attribute the proteolytic activity of the samples to either bromelain or papain, the IC_50_ values of both proteases were first established. Specifically, FIP and bromelain reference standards were used to define the typical IC_50_ for bromelain, whereas a papain standard was employed to determine the corresponding value for papain. While FIP and the commercial bromelain standard exhibited comparable IC_50_ values (0.012 and 0.016 µmol/mg, respectively), the IC_50_ of papain was significantly lower (0.0033 µmol/mg). Regarding the tested samples, BROMELAIN 1 and 2 displayed contrasting results (Table 2); specifically, the IC_50_ of BROMELAIN 1 (0.0153 µmol/mg) closely matched those of the bromelain references, whereas BROMELAIN 2 yielded 0.0065 µmol/mg, which is comparable to that of papain.

**Figure 1:**
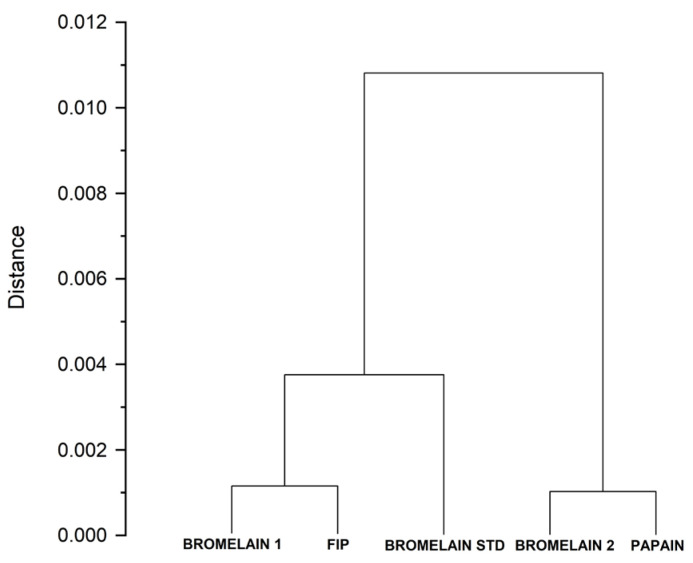
Univariate clustering analysis of the IC_50_ values. BROMELAIN 1 and bromelain reference samples are grouped in a distinct cluster compared to the group including BROMELAIN 2 and papain.

A univariate clustering analysis was performed to group the IC_50_ data into homogeneous clusters (Figure 1). This statistical evaluation revealed distinct sample profiles, grouping BROMELAIN 2 together with papain, while BROMELAIN 1 clustered with FIP and the standard bromelain. These findings confirm that the enzymatic activity reported in Table 1 is primarily attributable to bromelain in BROMELAIN 1, whereas the activity in BROMELAIN 2 is driven by a papain-like enzyme.

### 2.2. Purity and Characterization of Bromelain-Containing Ingredients

To further characterize the quality of each assessed sample, total protein concentrations for the tested formulations were first evaluated via BCA (bicinchoninic acid) assay, as shown in Table 3. While BROMELAIN 1 consisted almost entirely of protein (88.71%), the remaining samples exhibited markedly lower contents: 31.13% for BROMELAIN 2, and notably low levels for BROMELAIN 3 and 4 (3.26% and 3.78%, respectively).

**Figure 2:**
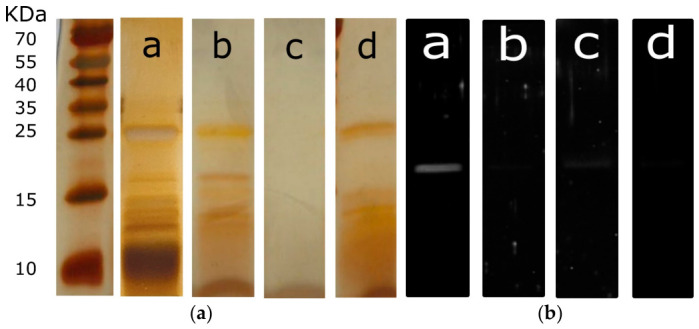
(**a**) Silver staining and (**b**) SDS-PAGE of the tested formulations. The first left lane shows the molecular weights labels; a, b, c, and d lanes refer to BROMELAIN 1, 2, 3, and 4, respectively.

The samples were analyzed using silver-stained SDS-PAGE to further assess their overall protein quality. Given that bromelain is inherently a multiprotein extract, this technique allows for a comprehensive comparison of the protein banding patterns and their corresponding molecular weights within each sample. As shown in Figure 2a, BROMELAIN 1 (lane a), BROMELAIN 2 (lane b), and BROMELAIN 4 (lane d) are characterized by a typical band at approximately 25 kDa—which likely corresponds to the main bromelain enzyme—alongside several bands of lower molecular weight [15]. Conversely, BROMELAIN 3 (lane c) shows no detectable bands, confirming that its lack of enzymatic activity is directly due to a complete absence of protein in the tested product.

Protein banding patterns were analyzed to evaluate the overall protein profiles of the samples and to specifically verify the presence or absence of the characteristic 25 kDa band, which corresponds to either bromelain or papain. The intensity of this band was quantified for each sample, and the results—expressed as the area normalized to the amount of loaded protein—are presented in Table 4. In contrast to the enzymatic activity data (Table 1), BROMELAIN 1 exhibited the highest band intensity (313.3 area/µg protein), followed by BROMELAIN 2 (201.3 area/µg protein) and BROMELAIN 4 (140.8 area/µg protein).

The presence of bands with molecular weights below 25 kDa is likely attributable to protein degradation fragments or other co-extracted proteins and peptides. This observation raised concerns regarding the purity of the tested samples. Consequently, protein purity was calculated as the ratio of the specific band areas to the total band area, where 100% represents pure bromelain. As shown in Table 5, the presence of these bands of lower molecular weight significantly reduces the calculated purity to 10.82% for BROMELAIN 1, 14.33% for BROMELAIN 2, and 17.15% for BROMELAIN 4.

To unequivocally confirm the presence and structural integrity of bromelain, the samples were subjected to Western blot analysis using a bromelain-specific antibody (Figure 2b). Although silver-stained SDS-PAGE revealed multiple bands within the samples (Figure 2a), only a single 25 kDa band exhibited immunoreactivity. This confirms the presence of intact, full-length bromelain and indicates an absence of autoproteolysis or bromelain-derived proteolytic fragments. Consequently, the lack of signal from the other bands observed in the silver stain demonstrates that they are unrelated to bromelain. Table 6 presents the quantification of the 25 kDa band, with the results expressed as the percentage of bromelain within the total ingredients and relative to the total protein content.

The bromelain content in BROMELAIN 1 is higher than the other three formulations, confirming that the measured activity is mainly due to the presence of bromelain enzyme. It is interesting to note that BROMELAIN 2 shows no bromelain-related signal despite having the highest enzymatic activity among the tested ingredients, equal to 3802.85 ± 138.87 GDU/g. The low or absent enzymatic activity in the samples of BROMELAIN 3 and 4 is confirmed by the absence of bromelain-related signals in the Western blot analysis.

**Figure 3:**
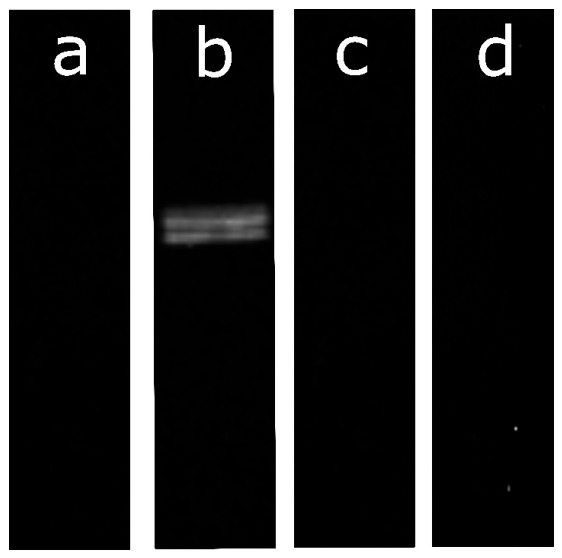
Western blot of the formulations. Lane a, b, c, and d show BROMELAIN 1, 2, 3, and 4, respectively.

To better investigate the potential source of enzymatic activity in BROMELAIN 2, a Western blot analysis with a papain-specific antibody was performed (Figure 3). While BROMELAIN 1 (lane a), 3 (lane c), and 4 (lane d) showed no papain-related signal, BROMELAIN 2 (lane b) showed a band related to papain, which indicates that the proteolytic activity observed is due to papain instead of bromelain.

These findings align with the IC_50_ value determined for BROMELAIN 2 (0.0065 ± 0.0001 µmol/mg) and its subsequent clustering with the papain standard. Consequently, this indicates that evaluating the IC_50_ serves as an effective method for ascertaining whether a sample’s proteolytic activity is driven by true bromelain or by other proteases such as papain.

## 3. Discussion

The adulteration of botanical ingredients, including bromelain and pineapple extracts, has raised significant concerns regarding their purity, efficacy, and safety. While monitoring the enzymatic activity of bromelain-based ingredients and dietary supplements serves as a valuable initial assessment of raw material quality, this approach alone is not sufficient to guarantee product authenticity. Enzymatic assays provide an essential but incomplete picture of bromelain authenticity; for instance, traditional methods based on gelatin digestion suffer from low sensitivity and lack specificity. Relying solely on these tests can lead to false-positive results, as alternative natural or synthetic proteases, such as papain, can produce comparable Gelatin Digestion Unit (GDU) values. Furthermore, bromelain is a complex mixture of proteins whose composition and catalytic properties vary depending on the plant source (e.g., fruit vs. stem) and the extraction method employed [16]. Indeed, enzymes derived from different anatomical parts of the pineapple exhibit distinct substrate specificities and proteolytic activities. Consequently, a robust quality assessment must involve evaluations of both the total enzymatic activity and the structural identity of the proteolytic components.

This work demonstrates that combining functional and structural analyses is essential for reliably authenticating bromelain in a real-world case study. Specifically, the proposed methodology combines the initial evaluation of overall enzymatic activity with its specific correlation via inhibition curves using a selective inhibitor. Alongside this functional assessment, silver-stained SDS-PAGE was utilized to characterize the complete protein profiles, while Western blotting was performed to definitively authenticate the presence of true bromelain in the extracts.

Our initial enzymatic assays revealed that only BROMELAIN 1 and 2 exhibited proteolytic activity comparable to their nominal values (Table 1), whereas BROMELAIN 3 and 4 displayed little to no activity. This functional deficit in BROMELAIN 3 and 4 was further corroborated by their negligible total protein content (via BCA assay) and the lack of protein bands for the BROMELAIN 3 sample on silver-stained SDS-PAGE.

Crucially, while BROMELAIN 2 met its declared enzymatic activity specifications, further molecular characterization revealed that this activity did not originate from bromelain. Western blot analysis using specific antibodies confirmed the presence of full-length, intact bromelain exclusively in BROMELAIN 1. By contrast, BROMELAIN 2 yielded no bromelain signal but tested positive for papain, demonstrating that its apparent compliance was due to substitution with an undeclared alternative protease. Ultimately, of the tested formulations, only BROMELAIN 1 met the specifications for both functional enzymatic activity and authentic bromelain content.

The identification of papain in BROMELAIN 2 highlights a clear instance of eco-nomically motivated adulteration. Papain, a protease derived from *Carica papaya* L., is generally more abundant, easier to extract in high yields, and less expensive than bromelain. Papain exhibits broad-spectrum proteolytic activity that can easily mimic bromelain in non-specific functional assays, such as the standard gelatin digestion method. This makes it an ideal, “silent” substitute for manufacturers whose raw materials are tested solely based on simple Gelatin-Digesting Unit (GDU) metrics. While specific, peer-reviewed reports detailing the exact substitution of bromelain with papain in commercial dietary supplements are notably scarce in the literature, the broader practice of replacing high-value botanical ingredients with cheaper biological proxies is well documented.

These findings validate our proposed analytical workflow. Specifically, the determination of IC_50_ values combined with univariate clustering proved to be a highly effective strategy for correlating total enzymatic activity with the specific protease responsible for it. The discrepancies uncovered in this study—ranging from the total absence of active proteins to the deceptive use of undeclared proteases—raise significant concerns regarding product integrity and consumer safety.

Furthermore, these results highlight a critical gap in current regulatory frameworks for enzyme-based food supplements. Although isolated national monographs exist (e.g., the Health Canada monograph for fruit bromelain), the lack of universally harmonized standards for multi-enzyme botanical preparations presents a major challenge for quality assurance. This regulatory vulnerability facilitates the growing incidence of botanical adulteration, a trend consistently documented by the Botanical Adulterants Prevention Program (BAPP) [17]. It is evident that standardizing a product based solely on overall proteolytic activity (e.g., GDU) is inadequate, as it allows for the erroneous validation of adulterated raw materials. To the best of our knowledge, this is the first investigation specifically addressing the adulteration of bromelain-containing nutraceuticals using a comprehensive, multi-analytical strategy in a real-world market context. We acknowledge that the relatively limited number of samples analyzed may not capture the full variability of the global dietary supplement market. However, despite the small sample size, the significance and validity of this work remain substantial. By highlighting clear quality discrepancies and blatant adulterations in commercially available products, our findings unequivocally demonstrate that current routine quality controls are insufficient. This research provides a robust multidisciplinary framework, integrating functional biochemical analyses with structural immunological techniques, that serves as a fundamental prerequisite for future investigations. Ultimately, adopting such an integrated approach is essential for regulatory authorities and manufacturers to ensure the authenticity, consistency, and safety of products, thereby restoring consumer confidence in plant-based nutraceuticals.

## 4. Materials and Methods

### 4.1. Samples

The bromelain samples used in this study include an International Pharmaceutical Federation (FIP) standard (FIP LGC Standard, Teddington, UK), a certified food-grade pineapple stem extract (Bromeyal^®^, Giellepi S.p.A., Milan, Italy) subsequently indicated as BROMELAIN 1, and three commercial bromelain ingredients manufactured in India, denominated as BROMELAIN 2, BROMELAIN 3, and BROMELAIN 4.

### 4.2. Evaluation of Bromelain Activity

The activity of bromelain was measured spectrophotometrically using a specific chromogenic substrate (pGlu-Phe-Leu-p-nitroanilide; Merck, Sigma-Aldrich, Darmastadt, Germany, cat. no. P3169) which, once cleaved, releases a yellow product that absorbs light at 410 nm. Bromelain activity was quantified by interpolating the absorbance of cleaved substrate against a calibration curve prepared with the FIP standard in the range 0–250 µg/mL. Results are expressed as GDU/g of formulation.

Briefly, a 0.1 mL aliquot of the FIP standard in a concentration range of 0 to 250 µg/mL or a 0.1 mL aliquot of the samples in a concentration range of 80 to 150 µg/mL was added to the corresponding test tube containing 1.25 mL of the pre-heated reaction mixture consisting of 0.1 M phosphate buffer at pH 6.5, 0.3 M KCl, 0.1 mM EDTA, and 3 mM DTT, as well as 0.15 mL of chromogenic substrate. Mixtures were incubated at 45 °C for 2 h under agitation at 400 rpm, and then the reaction was stopped by adding 0.1 mL of HCl 3 N. A negative control was also prepared with the same reaction mixture of samples, with 0.1 mL of HCl 3 N added to stop the reaction. A volume of 0.2 mL of the samples was transferred to a 96-multiwell transparent plate to record the absorbance at 410 and 750 nm. The analysis was performed in triplicate.

To evaluate the quality of bromelain and identify the possible presence of other proteases such as papain, the half-maximal inhibitory concentration (IC_50_) was determined by using a specific serine/cysteine protease inhibitor (antipain dihydrochloride from a microbial source, A6191, Merck Sigma-Aldrich, Darmstadt, Germany). The specific inhibitor was used at concentrations of 4, 2.6, 1.5, 1, 0.5, and 0 µM, and IC_50_ values were compared to those obtained for FIP, the bromelain standard (Merck Sigma-Aldrich B4882), and the papain standard (Merck Sigma-Aldrich P5306). IC50 values were then analyzed via a univariate clustering analysis with the OriginPro^®^ 2024 software (version 10.1) to group values based on similarities.

### 4.3. Total Protein Content

Total protein content in the formulations was evaluated via BCA protein assay, which is based on the usage of bicinchoninic acid (BCA, micro-BCA protein assay kit, 23235, Thermo Fisher Scientific, Waltham, MA, USA) for the colorimetric detection and quantification of total protein. This method combines the well-known reduction of Cu^2+^ to Cu^+^ by protein in an alkaline medium. The purple-colored reaction product of this assay is formed by chelating two molecules of BCA with one cuprous ion. This product can be quantified by reading the absorbance at 562 nm. Protein concentration was determined by interpolating the absorbance with a calibration curve prepared with BSA (bovine serum albumin standard, Thermo Fisher Scientific, 23209) (40, 30, 20, 10, and 2 ng/mL), and values are expressed as % in the formulation.

### 4.4. Evaluation of Bromelain Purity

Bromelain purity in the formulations was evaluated via SDS-PAGE silver staining and Western blot analysis for a specific antibody-mediated recognition. Samples were prepared as follows: 4 µL of Laemmli buffer (Merck Sigma-Aldrich) was added to 12 µL of the sample, vortexed, and boiled for 10 min at 95 °C to achieve protein denaturation. After centrifugation, samples were loaded into the gel and proteins were separated by electrophoresis under denaturing conditions using a 15% polyacrylamide gel (prepared with acrylamide/bisacrylamide 30%, Merck Sigma-Aldrich, A3699; Trizma base 1.5 M pH 8.8, Merck Sigma-Aldrich, T1503; sodium dodecyl sulfate 10%, Merck Sigma-Aldrich, RDD021; ammonium persulfate 10%, Merck Sigma-Aldrich, A3678; tetramethylethylenediamide, Merck Sigma-Aldrich, T9281; and water). Electrophoresis was performed in a running buffer (prepared with Trizma base; sodium dodecyl sulfate; and glycine, Merck Sigma-Aldrich, G8898) at 25 mA.

The SDS-PAGE was performed by loading 10 µg of the samples onto the gel. The pre-stained protein ladder (PageRuler™ Prestained Protein Ladder, 10 to 180 kDa, 26616, Life Technologies, Carlsbad, CA, USA) was used to determine the molecular weight of bands. Proteins were silver-stained with the following procedure. The gel was first incubated with a fixation solution (40% ethanol, 32221, Merck Sigma-Aldrich; 10% acetic acid, 1000632511, Merck Sigma-Aldrich; and 50% water) for 30 min, treated with a solution composed of 4 mM DTT in 20% ethanol, 5% acetic acid, and 75% water for 30 min, and rinsed in 0.5% potassium dichromate (207802, Merck Sigma-Aldrich) for 5 min and water for a further 5 min. The gel was equilibrated in 0.1% silver nitrate (S7276, Merck Sigma-Aldrich) for 30 min, rinsed with water, and incubated with the complex formation solution (0.02% paraformaldehyde, Merck Sigma-Aldrich, 16005; 3% sodium carbonate, Merck Sigma-Aldrich, S5761, at pH 12) until the protein bands were visible. The reaction was blocked by adding 1% acetic acid. The relative percentage of each band, including the one potentially corresponding to bromelain (molecular weight of 25 kDa), was calculated as follows:

(1) $$\;Band\;intensity\;\left(\%\right)\;=\;\frac{Band\;intensity}{Total\;intensity\;of\;bands}\;\times \;100$$

The intensity of the band potentially corresponding to bromelain is also expressed as the area/µg of proteins, using:

(2) $$\;Bromelain\;\left(area/µg\right)\;=\;\frac{Bromelain\;band\;area}{µg\;of\;protein\;loaded\;in\;gel}$$

Western blot analysis was performed to confirm bromelain-related bands. Bromelain standard B5144 was used to prepare a standard curve for quantification (25, 50, 75, and 100 ng), while samples were prepared as follows: BROMELAIN 1 at 80, 60, and 40 ng; BROMELAIN 2 at 100 and 120 ng; BROMELAIN 3 at 1296 ng (the maximum amount of protein possible); and BROMELAIN 4 at 500 and 1000 ng.

After SDS-PAGE electrophoresis, proteins were transferred to a nitrocellulose membrane (Merck Sigma-Aldrich GE10600001) using a transfer buffer (Trizma base, glycine, and methanol, Merck Sigma-Aldrich, 1037262002) at 25 mV overnight at 4 °C. The membrane was first incubated with 5% milk in TBS-T 1X (blocking solution) at room temperature (15–30 °C) for 1 h at 50 rpm and then with bromelain-specific antibody (AS09552, Agrisera, Vännäs, Sweden) and a secondary antibody (AB205718, Abcam, Cambridge, UK) for 2 h and 1 h at room temperature, respectively. Both anti-bodies were prepared in 5% milk. Bromelain was detected by chemiluminescence with an imaging system using a Western blotting substrate (Thermo Fisher Scientific, 10455145). Bromelain content was calculated by quantifying bands with the ImageJ 1.53a software. Briefly, the band densities were subtracted from the background and interpolated with the calibration curve obtained with the bromelain standard B5144. The amount was calculated as follows:

(3) $$\;Bromelain\;amount\;\left(ng\right)\;=\;\frac{y-q}{m}$$

where y is the band density, m is the angular coefficient, and q is the intercept.

The bromelain concentration (mg/mL) was calculated with:

(4) $$\;Bromelain\;contrantion\;\left(mg/mL\right)\;=\;\frac{Bromelain\;amount\;(ng)}{volume\;\;of\;sample\;loaded\;\left(µL\right)\;\times \;1000}$$

while the bromelain percentage in the formulation and in the proteins were calculated using:

(5) $$\;Bromelain\;on\;formulation\;\left(\%\right)\;=\;\frac{Bromelain\;amount\;(mg/mL)}{formulation\;concentration\;(\frac{mg}{mL})}\;\times \;100$$

(6) $$\;Bromelain\;on\;proteins\;\left(\%\right)\;=\;\frac{Bromelain\;amount\;(mg/mL)}{proteincon\;centration\;(\frac{mg}{mL})}\;\times \;100$$

To assess the presence of papain, Western blot analysis was performed with a papain polyclonal antibody (PA5-117633, Invitrogen, Carlsbad, CA, USA) and the secondary antibody (AB205718, Abcam). After antibody incubation at room temperature, detection was performed by chemiluminescence with an imaging system using a Western blotting substrate (Thermo Fisher Scientific, 10455145).

## 5. Conclusions

In conclusion, our findings suggest that implementing integrated analytical methods can be highly beneficial for the reliable validation of bromelain quality in dietary supplements. By combining functional enzymatic activity measurements with structural protein profiling and immunodetection, the authenticity, purity, and safety of bromelain-containing products can be more accurately evaluated. This comprehensive approach may facilitate regulatory compliance and help to protect consumer health, thereby supporting the overall integrity of the nutraceutical market. As product integrity is closely linked to both safety and efficacy, the potential presence of undeclared proteases or diluents may alter a product’s allergenicity, immunogenicity, or gastrointestinal effects, potentially posing risks to consumers. Therefore, moving toward stricter raw material specifications, such as verification of declared enzymatic activity, a minimum protein content, and identity confirmation via proteomics or immunodetection, could substantially enhance quality control frameworks, better aligning them with therapeutic and consumer safety objectives.

## Figures and Tables

**Table 1:** The total bromelain activity, expressed as GDU/g. Recovery refers to the percentage of the measured activity value of bromelain relative to the declared value. The results are expressed as mean values ± standard deviation of three experiments.

Formulation	Measured Value (GDU/g)	Declared Value (GDU/g)	Recovery (%)
BROMELAIN 1	2658.7 ± 133.7	2400	110.8
BROMELAIN 2	3802.8 ± 138.9	3090	123.1
BROMELAIN 3	<LOQ *	2400	N.D.
BROMELAIN 4	6.4 ± 0.1	2400	0.3

* LOQ = 0.065 GDU/g; N.D. = not detectable.

**Table 2:** IC_50_ values calculated from dose–response curves of inhibition activity. The results are expressed as mean values ± standard deviation.

Formulation	IC_50_ (µM)	IC_50_ (µmol/mg of Sample)
BROMELAIN 1	2.29 ± 0.04	0.0153 ± 0.0002
BROMELAIN 2	0.52 ± 0.01	0.0065 ± 0.0001
BROMELAIN 3	N.D.	N.D.
BROMELAIN 4	N.D.	N.D.
FIP ^1^ standard	1.87 ± 0.18	0.0120 ± 0.0010
Bromelain standard	1.57 ± 0.56	0.0160 ± 0.0060
Papain standard	0.33 ± 0.02	0.0033 ± 0.0002

^1^ FIP = International Pharmaceutical Federation standard; N.D. = not determinable due to the absence of proteolytic activity.

**Table 3:** Protein content in the formulations as determined by BCA1. The results are expressed as mean values ± standard deviation.

Formulation	Protein (% on Formulation) Measured by BCA ^1^ Assay
BROMELAIN 1	88.7 ± 10.5
BROMELAIN 2	31.1 ± 1.7
BROMELAIN 3	3.3 ± 0.2
BROMELAIN 4	3.8 ± 0.1

^1^ BCA= Bicinchoninic Acid protein assay.

**Table 4:** Quantification of the band at 25 kDa, expressed as the area normalized to the amount of loaded protein.

Formulation	Area/µg Proteins
BROMELAIN 1	313.3
BROMELAIN 2	201.3
BROMELAIN 3	N.D. ^1^
BROMELAIN 4	140.8

^1^ N.D. = not detectable.

**Table 5:** The relative percentage of the most representative bands detected. The percentage at 25 kDa corresponds to protein purity, defined as the ratio of the “specific band” area at 25 kDa to the total band area.

Molecular Weight (kDa)	BROMELAIN 1	BROMELAIN 2	BROMELAIN 3	BROMELAIN 4
25	10.8	14.3	N.D. ^1^	17.1
>15	N.D. ^1^	13.9	N.D. ^1^	N.D. ^1^
15	5.8	N.D. ^1^	N.D. ^1^	N.D. ^1^
<15	83.4	71.8	N.D. ^1^	82.9

^1^ N.D. = not detectable.

**Table 6:** Bromelain content measured with Western blot analysis. Results are expressed as bromelain (%) in the formulation and bromelain (%) in the proteins.

Formulation	Bromelain (% on Formulation)	Bromelain (% on Proteins)
BROMELAIN 1	8.500 ± 0.910	9.550 ± 1.020
BROMELAIN 2	0.040 ± 0.010	0.150 ± 0.050
BROMELAIN 3	0.003 ± 0.000	0.300 ± 0.015
BROMELAIN 4	0.020 ± 0.000	0.490 ± 0.120

## Data Availability

The original contributions presented in this study are included in the article. Further inquiries can be directed to the corresponding authors.

## Abbreviations

The following abbreviations are used in this manuscript:

GDU	Gelatin-Digesting Units
pNA	Z-Arg-Arg-p-nitroanilide
SDS-PAGE	Sodium Dodecyl Sulfate–Polyacrylamide Gel Electrophoresis
LOQ	Limit of quantification
ND	Not detectable
FIP	International Pharmaceutical Federation standard
IC50	Half-maximal inhibitory concentration
BCA	Bicinchoninic Acid protein assay
BAPP	Botanical Adulterants Prevention Program
